# Ruptured pulmonary artery pseudoaneurysm treated with stent graft: case report and literature review

**DOI:** 10.1186/s42155-022-00339-6

**Published:** 2022-11-22

**Authors:** Valère Barrot, Olivier Pellerin, Guillaume Reverdito, Marc Sapoval, Tom Boeken

**Affiliations:** 1 Assistance Publique - Hôpitaux de Paris Hôpital Européen Georges Pompidou Vascular and Oncological Interventional Radiology Department, 20 Rue Leblanc, 75015 Paris, France; 2 Université Paris Cité, Paris, France; 3 Assistance Publique - Hôpitaux de Paris Hôpital Européen Georges Pompidou, Radiology Department, Paris, France; 4 HeKA Team, INRIA, Paris, France

**Keywords:** Hemoptysis, Embolization, Aneurysm false, Pulmonary artery, Stent

## Abstract

**Background:**

Hemoptysis is a severe condition, associated with a high mortality rate from asphyxiation. Less than 5% of cases come from the pulmonary arterial circulation and large pseudoaneurysm are rarely treatable by stent graft.

**Case presentation:**

We present the case of a 74-year-old man who suffered from a new onset of hemoptysis despite a prior bronchial artery embolization. He underwent a rescue endovascular stent graft placement for a massive hemoptysis caused by a ruptured proximal pulmonary artery pseudoaneurysm. A short review of similar situations is provided.

**Conclusion:**

Salvage endovascular stent graft placement for a massive hemoptysis caused by a ruptured proximal pulmonary artery pseudoaneurysm is a viable salvage technique for life-threatening hemoptysis.

## Introduction

Severe hemoptysis is a serious condition, associated with a high mortality rate from asphyxiation (Parrot et al. 2018). A large study on the French nationwide database found that the most frequent etiology was lung cancer, followed by active tuberculosis and bronchiectasis (Fartoukh et al. 2021). Most cases are related to the bronchial arterial supply of lung (Yoon et al. 2002) and can be treated with bronchial artery embolization (BAE). Less than 5% of cases come from the pulmonary arterial circulation (Rabkin et al. 1987). In 2022, the CIRSE established new guidelines for BAE, where it recommends embolization for patients with life threatening or recurrent hemoptysis (Kettenbach et al. 2022). Technical success rates range from 90 to 100% and clinical success rates range from 82 to 100% after 24 h (Chun and Belli 2010; Dorji et al. 2021). Despite these high early success rates, the recurrence rate remains high (47%) (Heuvel et al. 2007); one of the reasons for recurrence is the implication of the pulmonary arterial circulation in the bleeding origin (Chun et al. 2010).

## Case report

**Fig. 1:**
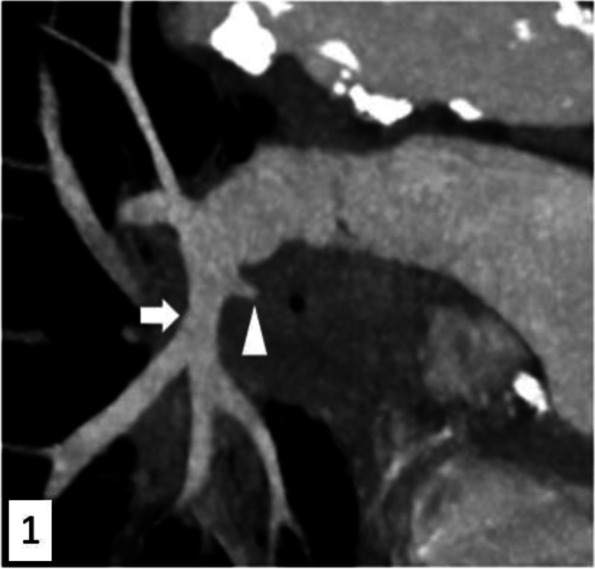
Treatment of a ruptured pulmonary artery pseudoaneurysm with a stent graft. Sagittal reconstruction of a computed tomography angiography before embolization shows a pseudoaneurysm (*arrow head*) arising from the left inferior pulmonary artery root (*arrow*). Sagittal MIP 10 mm reconstruction 80 keV 130 mAs Somatom X.Cite Siemens Healthineers Erlangen

A 74-year-old man treated for a stage IV lung adenocarcinoma initially presented with a mild hemoptysis (estimated volume: 100–200 mL over 24 h) and underwent a bronchial artery embolization at our institution. No complication was noted and the patient was discharged the day after. He presented with a severe recurrent hemoptysis one month later and was admitted in the intensive care unit for initial care. Nasal Oxygenotherapy was immediately administered at a 15 L/ minute flowrate for target oxygen saturation > 90%; invasive ventilation was considered unreasonable by the intensive care physician given the highly compromised health condition of the patient. Computed tomography angiography showed a pulmonary artery pseudoaneurysm arising from the left pulmonary artery adjacent to the lung tumor (Fig. 1), which was retrospectively not present one month prior to the recurrence. After a multidisciplinary discussion, a salvage treatment was proposed and the patient was transferred to the interventional radiology angiographic suite.

**Fig. 2:**
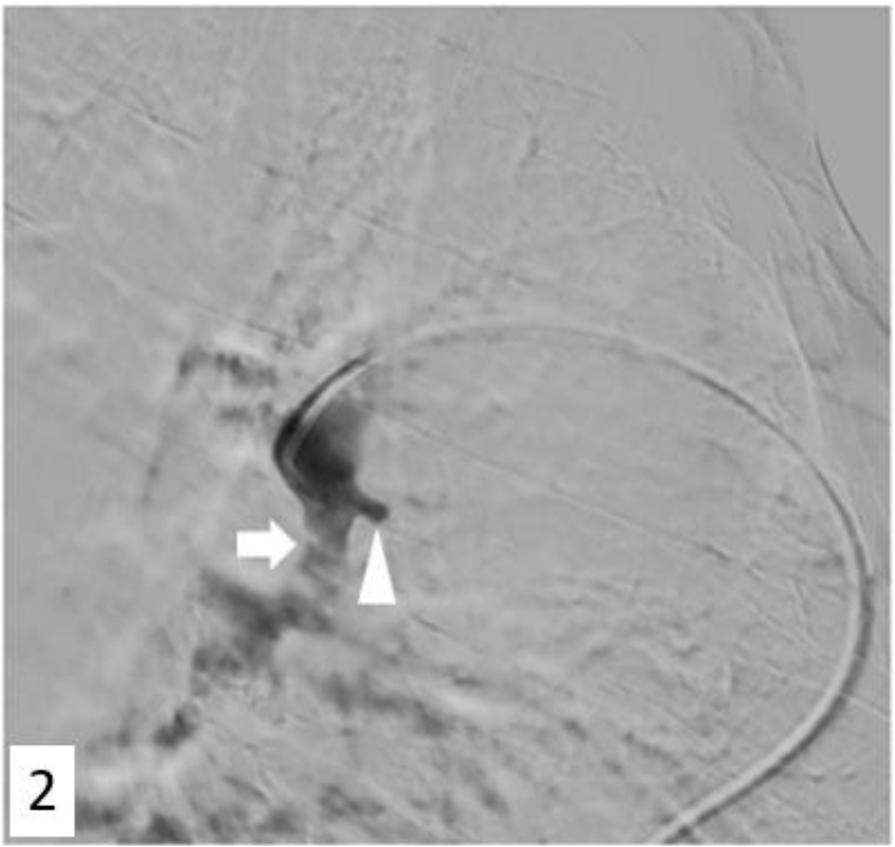
Left pulmonary artery angiography confirming the pseudoaneurysm location. Axiom Artis Q, Siemens Healthineers Erlangen

Access to the right femoral vein was performed using a 10-French vascular long sheath placed in the origin of the right atrium, under local anesthesia. The initial angiogram was performed in the left pulmonary artery using a pigtail 5-French catheter with an injection protocol of 30 mL of contrast injected at a rate of 15 mL/second. Cone-Beam computed tomography was not feasible given the overall patient agitation. Selective angiography within the inferior lobar artery using a multipurpose 5-French catheter confirmed the pseudoaneurysm arising from the posterior wall of the artery (Fig. 2). After careful selective catheterization of the pseudoaneurysm using a 2.4-French microcatheter, we injected a few mL of Onyx 34 (Medtronic) but the microcatheter instability did not allow us to inject enough to obtain a satisfactory filling. Subsequently an endovascular stent graft was deployed (11,5 × 33 mm LifeStream balloon expandable covered stent (Bard Medical)) to exclude the pseudoaneurysm. The stent was over dilated with the provided balloon at its origin to allow maximum coverage along its length and to prevent endoleak or material migration. The patient was immediately placed under an antiplatelet therapy (aspirin 75 mg daily).

**Fig. 3:**
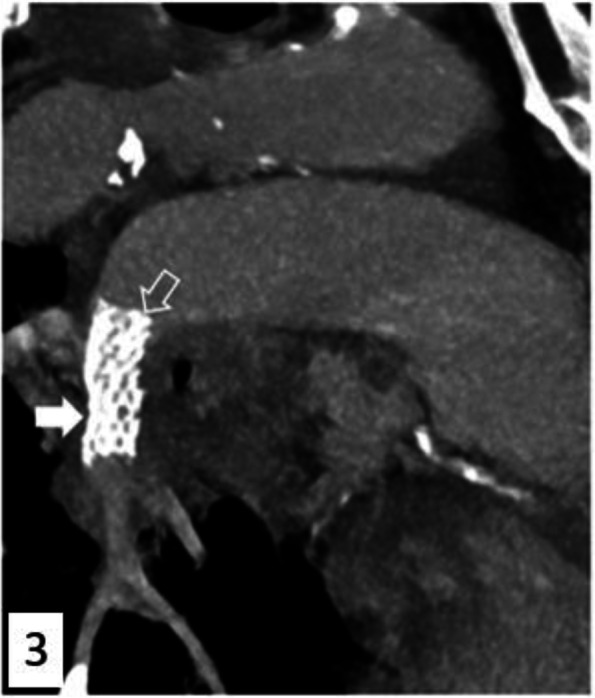
Postoperative computed tomography angiography after stent deployment (*empty arrow*) placement shows the exclusion of the pseudoaneurysm and the patency of downstream arteries. Sagittal MIP 10 mm reconstruction 80 keV 130 mAs Somatom X.Cite Siemens Healthineers Erlangen

Control angiography showed no immediate complication and confirmed the exclusion of the pseudoaneurysm, as reassessed by a second CT angiography (Fig. 3). There was no pulmonary infarction described on the postoperative scan. There were no acute complications and no recurrence during hospitalization and the patient was discharged on day 6. During the follow-up, the patient did not return to the hospital for an acute pathology. A CT scan performed one and a half months after this episode showed a permeable stent without pulmonary infarction.

## Discussion

Although the treatment of hemoptysis from the bronchial artery circulation is well known (Kettenbach et al. 2022), hemoptysis from the pulmonary artery circulation is a rare condition. Several case reports have been published regarding the treatment of pseudoaneurysms and various embolic materials have been used. Coils are the most common choice but may require the sacrifice of the feeding artery (O’Reilly et al. 2022; Yamakado et al. 2010; Law et al. 2022; Marcelin et al. 2018). This strategy is feasible if the pseudoaneurysm is relatively distal; however, in the present case, the target lesion was very proximal in the lobar artery. Onyx can be used in the pulmonary arterial circulation; Khalil et al. report a series of 12 cases (Khalil et al. 2012). Nevertheless, the use of such an embolic agent requires a narrow aneurysmal neck and the sacrifice of the parent artery in most cases. It was not possible in our case where the pseudoaneurysm originated from the trunk of the left pulmonary artery.

Covered stents allow the exclusion of the pseudoaneurysm while respecting the feeding artery (Klobuka and Short 2016; Park and Cwikiel 2007; Hannan et al. 2001; Wilson et al. 2000; Chou et al. 2006). The technical feasibility may be more complex due to the irregular caliber of the pulmonary arterial circulation, and could require proximal dilation to ensure optimal apposition of the stent graft against the arterial wall. In our case we used a femoral approach, although reports of distal embolization have been published using the brachial approach (Contegiacomo et al. 2021). Anecdotally, there are cases of pseudoaneurysms treated by direct percutaneous puncture and injection of thrombin or N-butyl cyanoacrylate (Lee et al. 2008). Marcelin and al (Marcelin et al. 2018) showed that embolization in the context of pulmonary artery hemoptysis was effective.

## Conclusion

In the absence of sufficient data, the choice of embolization material is left to the clinician. Importantly, pulmonary artery embolization is a viable salvage technique for life-threatening hemoptysis, particularly in proximal lesions, where sacrifice of the parent artery is not an option.

## Data Availability

The datasets used and analysed during the current study are available from the corresponding author on reasonable request.

## Abbreviations

BAE: Bronchial Artery Embolization
CIRSE: Cardiovascular and Interventional Radiological Society of Europe
CT: Computed Tomography
MIP: Maximum Intensity Projection
